# Large area MoS_2_ thin film growth by direct sulfurization

**DOI:** 10.1038/s41598-023-35596-5

**Published:** 2023-05-24

**Authors:** Kai-Yao Yang, Hong-Thai Nguyen, Yu-Ming Tsao, Sofya B. Artemkina, Vladimir E. Fedorov, Chien-Wei Huang, Hsiang-Chen Wang

**Affiliations:** 1 Division of Gastroenterology, Department of Internal Medicine, Kaohsiung Armed Forces General Hospital, 2, Zhongzheng 1st.Rd., Lingya District, Kaohsiung City, 80284 Taiwan; 2 Department of Mechanical Engineering, National Chung Cheng University, 168, University Rd., Min Hsiung, Chia Yi, 62102 Taiwan; 3 Nikolaev Institute of Inorganic Chemistry, Siberian Branch of Russian Academy of Sciences, Novosibirsk, Russia 630090; 4 Department of Natural Sciences, Novosibirsk State University, 1, Pirogova Str., Novosibirsk, Russia 630090; 5 Department of Nursing, Tajen University, 20, Weixin Rd., Yanpu Township, 90741 Pingtung County Taiwan; 6 Director of Technology Development, Hitspectra Intelligent Technology Co., Ltd., 4F., No. 2, Fuxing 4th Rd., Qianzhen Dist., Kaohsiung City, 80661 Taiwan

**Keywords:** Materials science, Nanoscience and technology

## Abstract

In this study, we present the growth of monolayer MoS_2_ (molybdenum disulfide) film. Mo (molybdenum) film was formed on a sapphire substrate through e-beam evaporation, and triangular MoS_2_ film was grown by direct sulfurization. First, the growth of MoS_2_ was observed under an optical microscope. The number of MoS_2_ layers was analyzed by Raman spectrum, atomic force microscope (AFM), and photoluminescence spectroscopy (PL) measurement. Different sapphire substrate regions have different growth conditions of MoS_2_. The growth of MoS_2_ is optimized by controlling the amount and location of precursors, adjusting the appropriate growing temperature and time, and establishing proper ventilation. Experimental results show the successful growth of a large-area single-layer MoS_2_ on a sapphire substrate through direct sulfurization under a suitable environment. The thickness of the MoS_2_ film determined by AFM measurement is about 0.73 nm. The peak difference between the Raman measurement shift of 386 and 405 cm^−1^ is 19.1 cm^−1^, and the peak of PL measurement is about 677 nm, which is converted into energy of 1.83 eV, which is the size of the direct energy gap of the MoS_2_ thin film. The results verify the distribution of the number of grown layers. Based on the observation of the optical microscope (OM) images, MoS_2_ continuously grows from a single layer of discretely distributed triangular single-crystal grains into a single-layer large-area MoS_2_ film. This work provides a reference for growing MoS_2_ in a large area. We expect to apply this structure to various heterojunctions, sensors, solar cells, and thin-film transistors.

## Introduction

Two-dimensional layered material MoS_2_ with atomically thick layers is one of the most common transition metal dichalcogenides (TMDs)^1–4^; it has an indirect energy gap of 1.2 eV in the bulk MoS_2_ semiconductor and a direct energy gap of 1.8 eV in the monolayer MoS_2_^5–9^. Single-layer TMDs has an excellent current switch ratio (on/off current ratio) in field effect transistors because of its direct energy gap^10,11^. These advantages can only be possessed by materials with atomic thickness^12^. MoS_2_ is a layered structure, which has good lubricity, pressure resistance, and wear resistance. It is mostly used in solid lubricants as well as in high-speed, heavy-duty, high-temperature, and chemical corrosion conditions^13–17^. This material has many potential applications, such as in field effect transistors, electronic devices, light-emitting diodes, sensors, and so on, due to its excellent optoelectronic properties^11,18–25^. In recent years, it has been found that MoS_2_ has semiconductor properties and can exist in the form of a single layer or a few layers^26^. Therefore, two-dimensional materials are widely discussed and studied by scientists. Many methods, including mechanical exfoliation^12,27–30^, thermally decomposed ammonium thiomolybdate^31–34^, sulfurization of Mo/MoO_3_^35^, and chemical vapor deposition (CVD)^36–42^, can be used to synthesize continuous MoS_2_ films. These methods are capable of producing many good quality MoS_2_ layers, however, achieving large area MoS_2_ thin films is challenging. The reason is that MoS_2_ tends to transform into nanoparticle and nanotube structures, leading to inefficient production in homogeneous synthesis and large-area MoS_2_ thin films, making it challenging to deploy production for electronic devices. Therefore, synthesizing large-area MoS_2_ thin films has attracted much research attention.

The growing method of this study is the direct sulfidation using molydenum/molybdenum oxide which also proposed by Lin et al.^35^ in 2012. The main process is to perform direct sulfurization reaction on a substrate coated with molybdenum oxide to obtain a MoS_2_ thin film. Molybdenum trioxide (MoO_3_) of about 3.6 nm is plated on a sapphire substrate and grown through two stages of heating. In the first stage, the heating time is one hour. The sample is placed into a furnace tube at 500 °C and passed through Ar/H_2_ mixed gas (Ar:H_2_ = 4:1) under controlled pressure of 1 Torr to convert MoO_3_ into MoS_2_. The reaction equation is as follows:

1 $${\text{MoO}}_{{3}} \left( {\text{s}} \right) \, + {\text{ H}}_{{2}} \left( {\text{g}} \right) \, \to {\text{ MoO}}_{{2}} + {\text{ H}}_{{2}} {\text{O}}\left( {\text{g}} \right),$$

In the second stage, the heating time is 30 min. Sulfur powder is placed into a tube first, and Ar/H_2_ mixed gas is introduced at 1000 °C. The pressure is controlled at 600 Torr. The MoO_2_ in the first stage reacts with sulfur vapor inside the tube to form MoS_2_. The reaction equation is as follows:

2 $${\text{MoO}}_{{2}} \left( {\text{s}} \right) \, + {\text{ 2S}}\left( {\text{g}} \right) \, \to {\text{ MoS}}_{{2}} \left( {\text{s}} \right) \, + {\text{ O}}_{{2}} \left( {\text{g}} \right),$$

The advantage of the direct sulfurization of molybdenum oxide is that it can grow a large area of MoS_2_ film and is similar to two-stage thermal decomposition.

In 2014, Choudhary et al.^43^ ref proposed a large-scale synthesis of MoS_2_ on a Si/SiO_2_ substrate using a two-step sputtering CVD method. The first step involved the deposition of Mo thin films on the substrate through the sputtering of Mo metal. In the second step, low-pressure CVD was employed to synthesize MoS_2_. Argon gas was used to transport sulfur into the reactor, where it reacted with the Mo film. Upon annealing at a high temperature of 600 °C and subsequent cooling to room temperature, the Mo films were successfully converted to MoS_2_ films. The results showed that this method effectively enabled the synthesis of large-area MoS_2_ from Mo thin films, and the thickness of the synthesized MoS_2_ films could be modulated by varying the sputtering times. The aforementioned studies indicate that large-area MoS_2_ thin films can be synthesized using a CVD process. This involves exposing a thin film containing Mo to sulfur powder in an Argon gas environment and subjecting the sample to high temperature conditions. The results demonstrate that this approach can yield MoS_2_ thin films with a substantial surface area.

In this study, we present the synthesis of MoS_2_ thin films through a CVD process. Our findings indicate that a Mo thin film can be converted into a MoS_2_ thin film using this method. Raman mapping, AFM, and high-resolution transmission electron microscopy (HRTEM) images confirmed the presence of a monolayer on the 2-inch sapphire wafer. Compared with MoS_2_ films grown on Si substrates, electrical measurements of sapphire substrates growth revealed p-type semiconducting behavior with remarkably high electron mobility and on–off ratio. This approach is compatible with conventional semiconductor processes and can be extended to other TMDs and arbitrary substrates by transferring MoS_2_.

## Materials and Methods

### Experimental materials and drug specifications of MoS_2_

A double-side polished sapphire substrate with a thickness of 300 nm was utilized for the growth of MoS_2_. The substrate was pre-coated with a 5 Å MoO_3_ film through sputter deposition. Subsequently, direct sulfidation was performed using sulfur powder (S) of 99.98% purity. The purpose is to avoid impurity residues that affect chemical vapor deposition or single-crystal residue impurities. The quartz tube and ceramic crucible in the high-temperature furnace tube (Tubular thermal) are cleaned with aqua regia. The concentrations of nitric acid and hydrochloric acid in aqua regia are 37% and 68–69%, respectively, to avoid impurities from the previous growth remaining in the cleaning process.

### Process and steps of growing MoS_2_ by direct sulfurization

**Figure 1:**
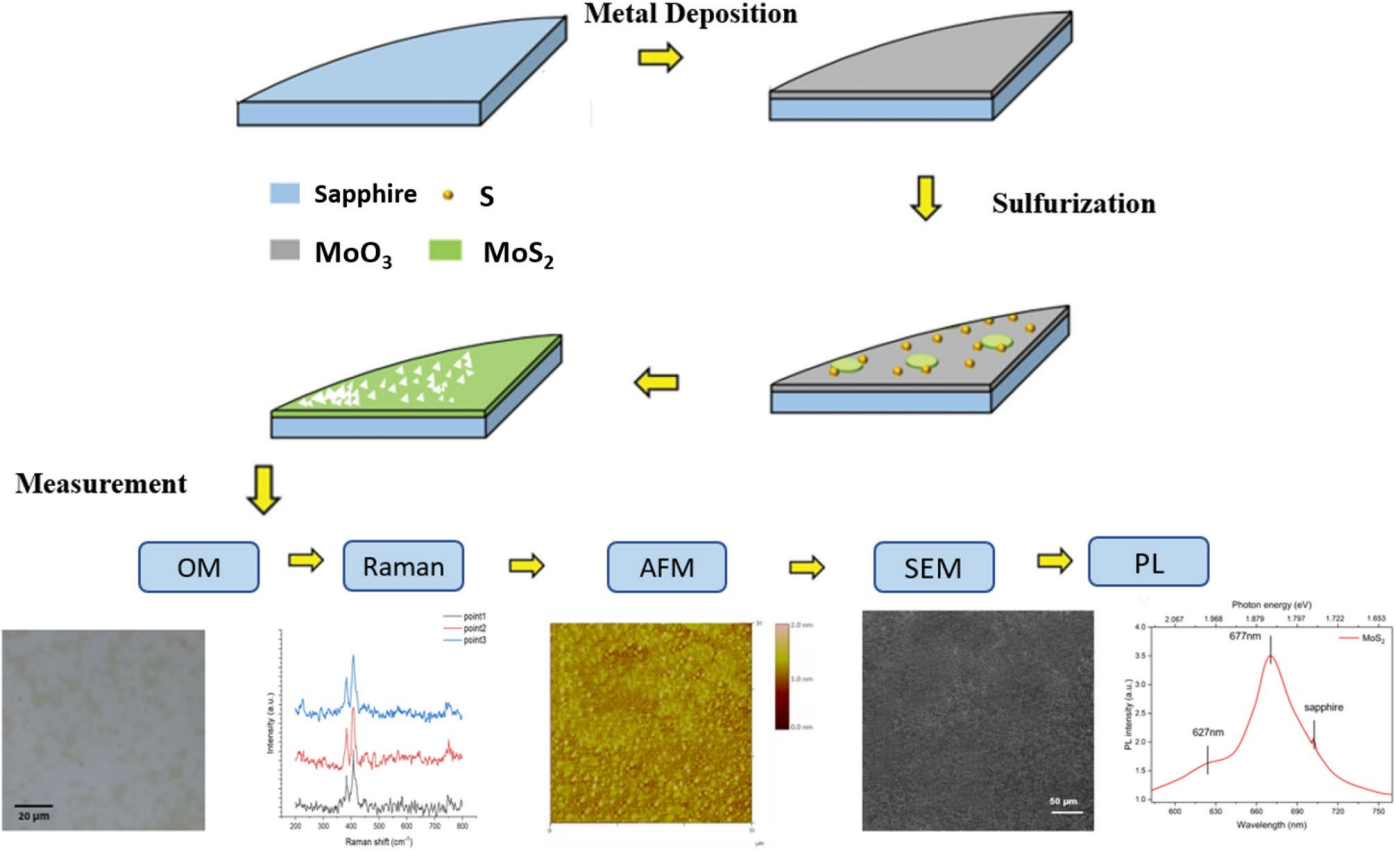
The flow chart of this study, starting with the growth of the material, followed by the optical measurement, and finally the measurement and analysis of the material properties.

The process of this study is shown in Fig. 1, a 2-inch sapphire substrate with 300 nm thickness pre-deposited 5 Å MoO_3_ was first prepared, cut into four equal parts, and cleaned with ultrasonic vibration in acetone for 10 min to remove surface impurities and oil stains and achieve a clean surface. Deionized water was used to remove organic solvent-acetone in ultrasonic vibration for 10 min. The sample was collected, and a high-pressure nitrogen gun was used to remove the moisture on the surface of the substrate. Direct sulfurization was used to grow MoS_2_. The substrate was placed under specific pressure and temperature conditions. In the furnace tube system, argon was used as the carrier gas to perform chemical reactions on the surface of the substrate by hot steam vulcanization to generate high-quality, large-area films. The application of direct sulfurization method in the preparation of single-layer TMDs started with the growth of MoS_2_. Initially, 500 sccm of argon gas was introduced to clean the interior of the system. The cavity was kept in a clean environment, and excess water vapor and oxygen in the quartz tube were removed for 10 min. The sample was grown with an argon flow rate of 400 sccm at a heating rate of 30 °C per minute. The growth temperature was 750 °C, which was maintained for 5 min. After the growth was completed, the gas volume was slowly decreased to  − 40 sccm per minute to reduce the damage of the newly formed MoS_2_ by airflow. The cover was opened once the temperature decreased to 400 °C. After opening the cover, the temperature was reduced to room temperature, and MoS_2_ structure was obtained at high temperatures. A 1/4 two-inch sapphire substrate with 1.2 g of sulfur powder (S) as a precursor was placed on a crucible in a furnace tube. The vaporization point of Mo is above 650 °C and the vaporization point of S is above 200 °C; as such, Mo in the gaseous phase under a high-temperature environment will undergo a chemical reaction to produce molybdenum oxide (MoO_3*−x*_) intermediate. The produced molybdenum oxide intermediate will diffuse to the substrate and react with vaporized sulfur to form MoS_2_ film. The distance between the substrate and sulfur layer is 24 cm. The single-layer large-area MoS_2_ can be effectively prepared by direct sulfurization. This method not only grows high-quality single-crystal materials but also prepares a large-area uniformly distributed film, which is beneficial to subsequent optoelectronic component manufacturing.

## Results

### Optical microscope analysis

**Figure 2:**
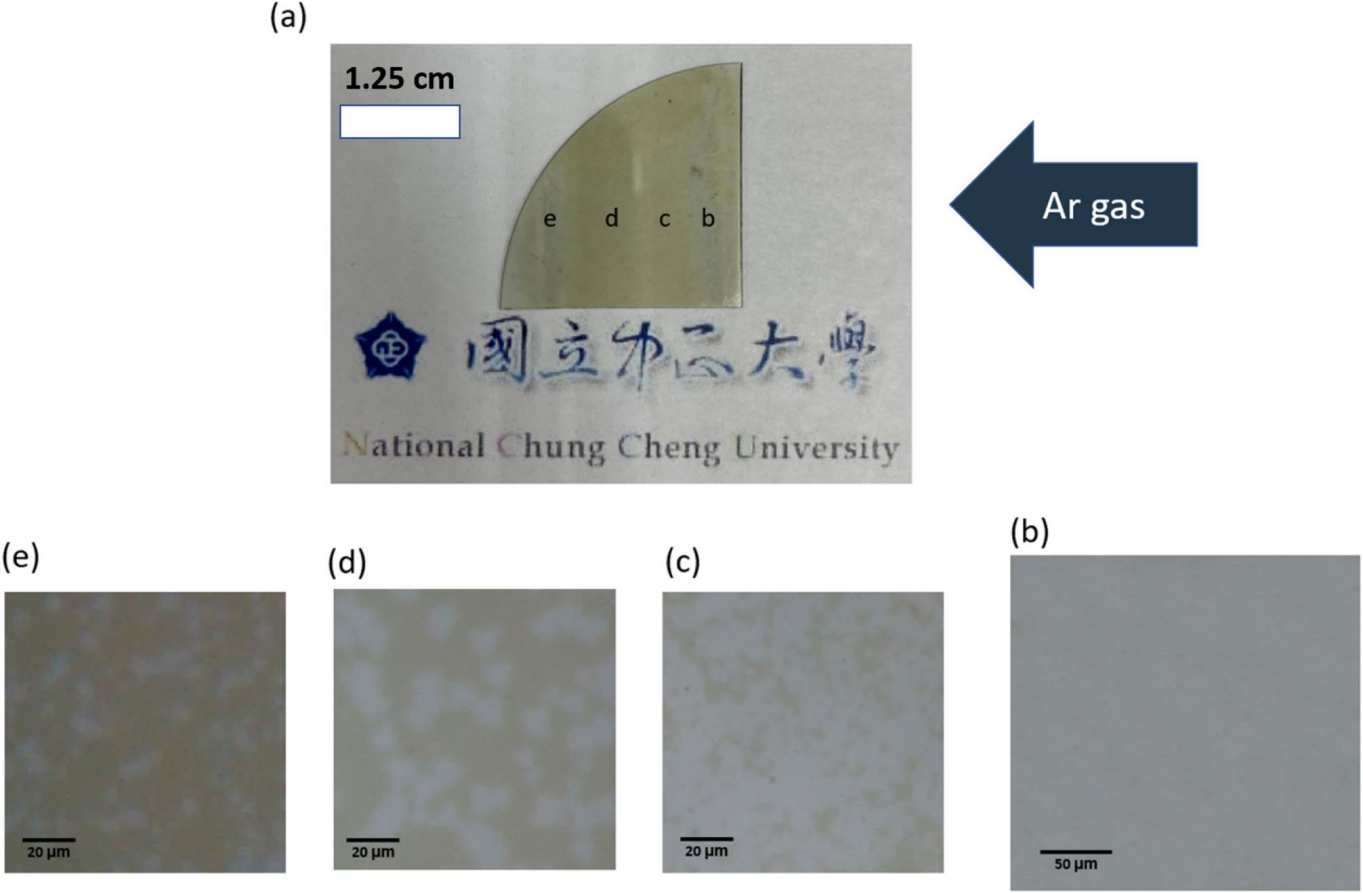
(**a**) Image of the sapphire substrate after the MoS_2_ growth is completed, (**b**–**e**) The OM images at positions (**b**–**e**) correspond to the positions indicated on the substrate in (**a**).

Under the optical microscope, the growth types of MoS_2_ have different shape distributions on the substrate. Figure 2 shows the optical microscope (OM) of MoS_2_ grown on the sapphire substrate. Figure 2a shows the substrate after actual growth, where the blue arrow indicates the direction of argon gas flow during CVD. The positions b, c, d, and e in the image present the growth distributions of different MoS_2_ shapes; Fig. 2b shows the OM image at position b in Fig. 2a, in which we found a single layer of MoS_2_ grown on the substrate in a large area, covering a larger area and almost completely covering the sapphire substrate. Figure 2c shows the OM image at position c in Fig. 2a, where the growth of single-layer MoS_2_ is not as good as that at position b, but single-layer MoS_2_ covers a larger area. Figure 2d shows the OM image at position d in Fig. 2a, in which the monolayer MoS_2_ grows irregularly on the sapphire substrate and has a light blue irregular shape distribution, indicating the existence of monolayer MoS_2_. Figure 2e is the OM image at position e in Fig. 2a, where the gray area is the sapphire substrate, and the scattered blue area is the area where MoS_2_ grows.

### SEM analysis

**Figure 3:**
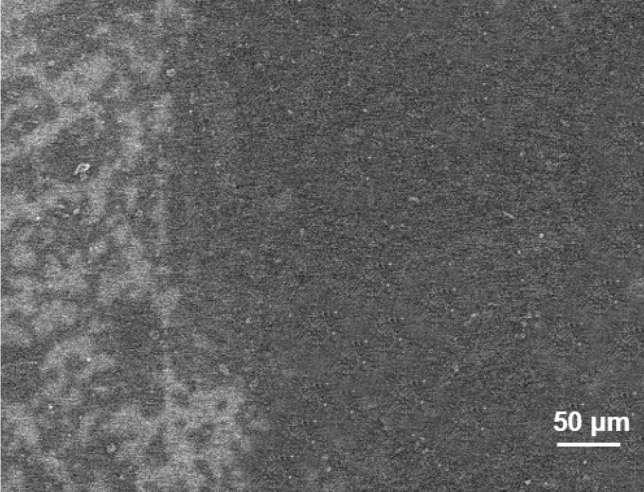
SEM image of junction between large area MoS_2_ and scattered MoS_2_ growth on sapphire substrate.

**Figure 4:**
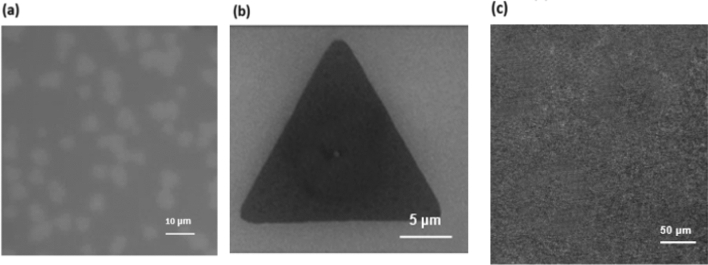
(**a**) OM and (**b**) SEM image of MoS_2_ grown by direct sulfurization method (**c**) SEM images of large-area MoS_2_ thin films in a large area.

We analyzed the growth of single-layer MoS_2_ thin film. Figure 3 shows the SEM image of junction between large area MoS_2_ and scattered MoS_2_ grown on the sapphire substrate. The size of MoS_2_ in the scattered area is about 20–30 μm. In the OM image, we can see uniform triangular or star-shaped two-dimensional structured MoS_2_ (Fig. 4a). Figure 4b shows a MoS_2_ SEM image that is single-layer and complete triangular. Taking a triangle as an example, Fig. 4c shows the SEM image of a large-area MoS_2_ thin film.

### Raman spectroscopy analysis

**Figure 5:**
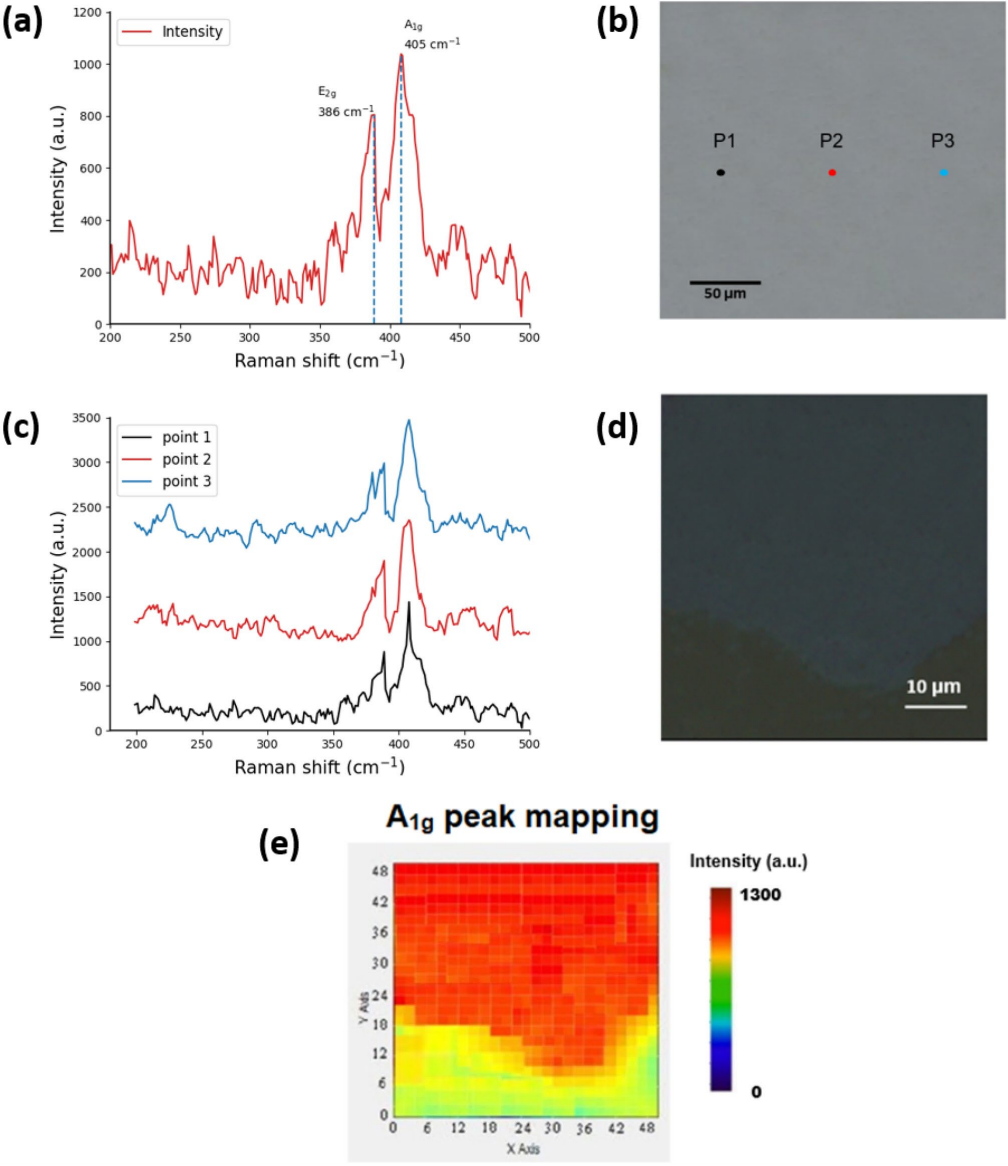
(**a**) Raman spectrum measurement map of a single layer (**b**) Raman spectrum measurement point schematic diagram (**c**) Raman spectrum measurement map of different selected areas (**d**) OM image of the selected Raman mapping range, (**e**) is the Raman mapping diagram of A_1g_.

We performed Raman measurement on the sapphire substrate sample grown with MoS_2_. A laser with a wavelength of 532 nm was used as the excitation light source. The X-axis in the spectrum represents the Raman shift, and the Y-axis represents the Raman intensity. Figure 5a shows the Raman measurement map of the scattered MoS_2_ in the region e of the OM image of MoS_2_ in Fig. 2a. We take the black, red, and blue points inside to measure the large-area growth as shown in Fig. 5b. Figure 5c shows the Raman measurement map of large-area MoS_2_ corresponding to the three black, red, and blue points in Fig. 5b.

The Raman shift is between 386.2 and 405.3 cm^−1^, and the value of peak difference (Δk) is about 19.1 cm^−1^. The results show that the large-area growth regions are all monolayers. Figure 5d shows the selected OM image of the Raman mapping range. Figure 5e presents the out-of-plane (A_1g_) Raman mapping image of Fig. 5d. The sapphire substrate is identified as the green portion. The distribution of single-layer MoS_2_ exhibits a relatively flat profile ranging from light green to yellow, and progressing towards orange-red on the graph. The mapping size of 50 μm × 50 μm shows a limited sampling area, yet the distribution remains consistently flat. This is further verified through Raman measurement, which demonstrates a uniform distribution of single-layer MoS_2_. These findings highlight the potential of MoS_2_ as a suitable material for the production of semiconductor devices.

### Atomic force microscopy analysis

**Figure 6:**
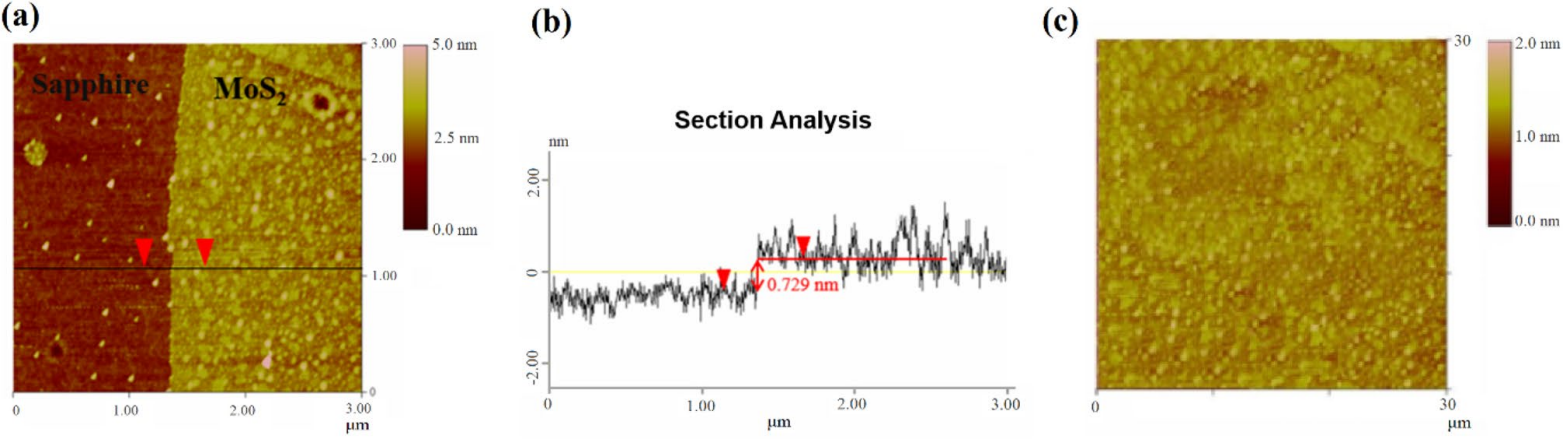
AFM image of MoS_2_, (**a**) is the surface morphology of sapphire substrate and MoS_2_, (**b**) is the height profile result of (**a**) cross-section, (**c**) is the surface morphology of large-area MoS_2_.

The thickness of the MoS_2_ thin film was determined through AFM measurement. The surface morphology and height profile results are shown in Fig. 6a,b, respectively. In Fig. 6a, the surface of the sapphire substrate and MoS_2_ can be clearly seen. Figure 6b shows that the thickness of the grown MoS_2_ on the sapphire substrate is 0.72 nm from the height profile of the cross-sectional line in Fig. 6a. This result indicates the successful synthesis of a single-layer MoS_2_ structure. In Fig. 6b, there is no substantial difference in profile height between the MoS_2_ layers or between the MoS_2_ layer and the sapphire surface. The average roughness of MoS_2_ films, as measured by AFM, ranges from 0.55 to 2.35 nm. It is important to note that AFM images depict the average distances of atoms on the sample surface, rather than the individual atoms themselves. In Fig. 6c, the 30 μm × 30 μm surface topography map of MoS_2_ displays a relatively uniform distribution of molecules, as indicated by the color bar. However, a slight asymmetry in distribution is also observed, suggesting that the MoS_2_ surface has a relatively smooth state.

### PL spectral analysis

**Figure 7:**
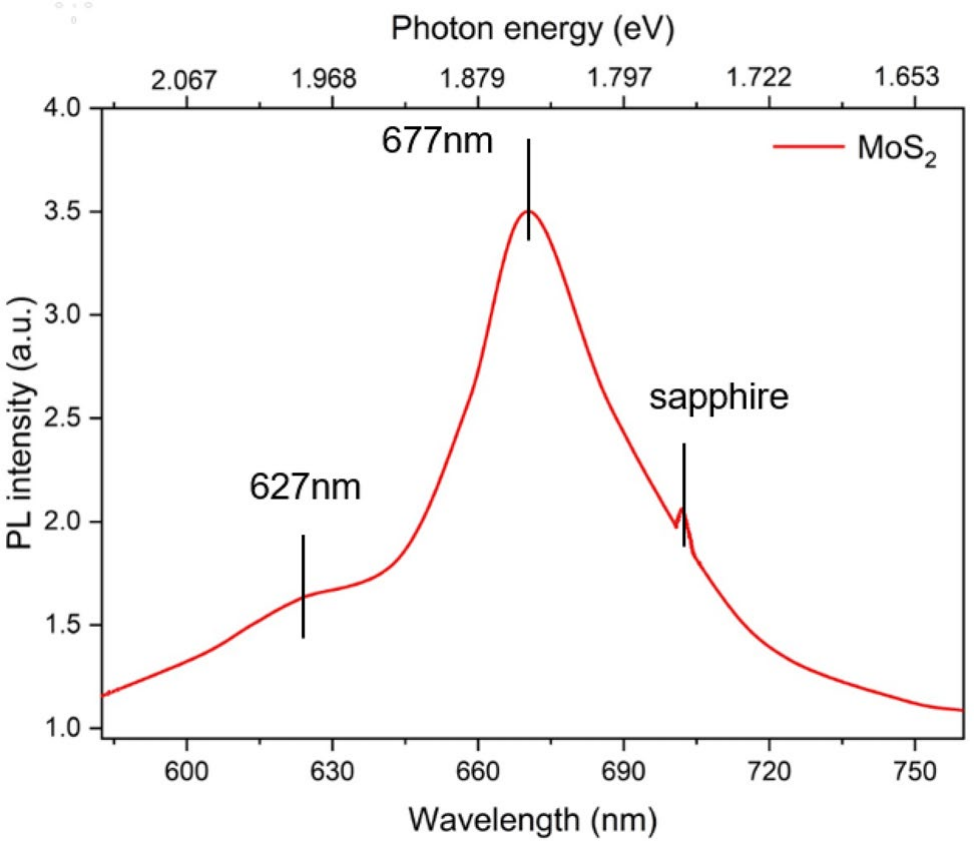
PL measurement results of monolayer MoS_2_, the luminescence band is at 677 nm, and the conversion energy is 1.83 eV.

Figure 7 shows the PL emission spectrum of MoS_2_. In the measurement, the 532 nm band was used as the laser excitation light source. The spectrum consists of two peaks, corresponding to A1 and B1 excitons at 677 and 627 nm, respectively. Both excitons have a direct excitonic transition from the valence band spin-orbital coupling energy separation^44^. The direct excitonic transition of the two resonances is the K point in the Brillouin zone. In general, the peak of MoS_2_ luminescence is about 67 nm, and the converted energy is 1.86 eV, which is the size of the direct energy gap of the MoS_2_ film. Based on the above data, the MoS_2_ grown has a single-layer structure.

## Discussions

In 2017, Zhu et al. reported the presence of two types of seed centers in the growth mechanism of MoS_2_, namely: (1) molybdenum-oxysulfide (MoO_x_S_*2-y*_) nanoparticles or nanocrystals for growing multilayer MoS_2_ and (2) two main edge-terminated irregular polygons for the growth of monolayer MoS_2_, the S-Mo Klein edge and the Mo zigzag edge, which appear roughly in equal quantities^45^. The morphology then evolves into a nearly triangular shape with predominant Mo meandering edges. Using a CVD system to grow MoS_2_, the impact of gas flow rate on MoS_2_ growth can be studied. The Ar flow rate was varied between 200 and 500 sccm. Results show that a faster flow rate mainly produced single layers, while a slower flow rate resulted in more layers. When the deposition rate exceeds the growth rate of MoS_2_, S-dominant growth occurs, which is the mechanism for monolayer MoS_2_ growth. Conversely, when the deposition rate is slower than the growth rate of MoS_2_, the dominant growth is molybdenum-oxysulfide, which is the mechanism for minority layer MoS_2_ growth.

In 2018, Zhou et al. further investigated the seed crystals responsible for the two growth mechanisms and the impact of temperature, in addition to gas flow rate, on the deposition rate^46^. The study's experimental results revealed that temperature with a central nucleation point played a significant role in the formation of the seed crystals.

In the same time, Taheri et al. shown that a three-stage mechanism was proposed for the growth of MoS_2_^47^. The first stage involves the evaporation of MoO_3_, which may be facilitated by the presence of sulfur. This can either result in the partial reduction of MoO_3_ to form MoS_2_ or the creation of molybdenum oxysulfide, both of which are volatile in nature. The evaporated molybdenum oxide then diffuses to regions on the substrate with lower molybdenum concentrations, as a result of the chemical potential difference. The experimental results by Taheri et al. revealed that under the conditions used in their study, diffusion lengths can attain values in the tens of micrometers range. This process plays a role in mitigating local morphological changes at the nanoscale within the precursor MoO_3_ films. In other words, the precursor film grows uniformly on the substrate as a local source of molybdenum, but does not dictate the ultimate morphology of the final MoS_2_. The second stage entails sulfidation to produce MoS_2_ through processes such as substitution of sulfur, lattice reorganization, and material redistribution. This leads to the uniform formation of single-crystal triangles of MoS_2_ across the substrate. The third stage involves the growth and merging of these triangles to form continuous MoS_2_. During this stage, MoS_2_ nanoparticles evaporate and are redeposited along the edges of the discrete triangles. The high density of triangles and long diffusion length allow for easy merging of the triangles, resulting in the filling of any gaps and the formation of continuous triangles with minimal overgrowth and voids. Meanwhile, the nanoparticles undergo evaporation and are not consumed. Unlike the growth process of CVD, there are no specific nucleation sites that cause morphological changes in MoS_2_. Instead, the process of vapor diffusion and nucleation under sulfidation occurs uniformly and locally at the microscale across the entire substrate, resulting in continuous MoS_2_ coverage. A unique aspect of the synthesis process is the cessation of carrier gas flow during the cooling step. Although MoS_2_ has a melting temperature of 1185 °C, its vapor pressure is small but not negligible at temperatures above 700 °C. By stopping the flow of the carrier gas, the trapped MoS_2_ vapor remains in equilibrium with the grown MoS_2_ crystals on the substrate for a longer period, thus preventing further evaporation of the film. Controlled experiments conducted by Taheri et al. showed that partial retention of MoS_2_ was achieved after annealing at 700 °C for 30 min in the presence of sulfur. Conversely, continuous air flow during cooling will cause the slow evaporation of MoS_2_, leading to its gradual loss and resulting in the formation of discrete triangular islands. This was confirmed by the experiment where complete evaporation of MoS_2_ was observed after only 5 min of annealing at 650 °C in the presence of nitrogen flow. These results strongly support the role of sulfur vapor in the growth of continuous MoS_2_. According to the flow direction of Argon gas, the growth of MoS_2_ progresses from a large coverage area to a smaller one, from fragmentation to large-area growth. By avoiding low-pressure growth, the process in a low-pressure environment minimizes unnecessary gas-phase reactions, which can lead to uneven distribution of gasified sulfur atoms. The resultant differences in the concentration of sulfur atoms can result in changes in the morphology of MoS_2_ from fragmentation to a more uniform, large-area distribution.

The Raman spectroscopy measurement showed that the grown MoS_2_ exhibits the characteristics of a monolayer. Raman mapping is the best method for analyzing the number of layers^48–50^. The PL spectral analysis results indicated that the distribution of the grown MoS_2_ monolayer is uniform, as demonstrated by the mapping diagrams obtained with the excitation light peak at 627 nm or 677 nm. The thickness of the sapphire substrate with periodic growth of MoS_2_ was analyzed using AFM. The selected area was confirmed to be a single layer, consistent with the results obtained from the Raman and PL spectroscopy analysis.

## Conclusions

The research can be divided into two parts. In the first part, we refer to the relevant literature and successfully grow large-area MoS_2_ in some areas of the sapphire substrate by changing the parameters through direct sulfurization. The single-layer thin film successfully overcomes the limitations of growing a large-area single-layer MoS_2_ thin film in chemical vapor deposition. In addition to adjusting the parameters, the method can achieve a continuous thin film of about 500 μm × 500 μm. The second part is the analysis of the results of Raman spectroscopy, photoluminescence spectroscopy, atomic force microscopy, and scanning electron microscopy. The large-area MoS_2_ film is composed of scattered triangular MoS_2_. The MoS_2_ film grown has monolayers. In summary, desirable modulated layers with a large area of MoS_2_ can be achieved by growth techniques using sulfided pre-deposited transition metals through sequential transition metal deposition and direct sulfurization.

Future works should grow large-area single-layer MoS_2_ thin films by direct sulfurization to develop MoS_2_ thin film for field effect transistors. In the past, semiconductors used three-dimensional materials, and the physical properties and device structures were developed to the 3-nm node. The present research used two-dimensional materials, with thickness less than 1 nm (1–3 layers of atomic thickness), which is closer to the limit of the thickness of solid semiconductor materials. The monolayer disulfide has a direct energy gap, making it suitable for application to solar cells. Previous works also used this characteristic to make solar cells with p-type materials, but many studies are limited by the size of MoS_2_ films. The present study contributes to knowledge on large-area growth to overcome existing problems and improve the efficiency performance of power generation.

## Data Availability

The datasets used and/or analyzed during the current study available from the corresponding author on reasonable request.
